# Optical Characterization of Ciprofloxacin Photolytic Degradation by UV-Pulsed Laser Radiation

**DOI:** 10.3390/molecules26082324

**Published:** 2021-04-16

**Authors:** Tatiana Tozar, Mihai Boni, Angela Staicu, Mihail Lucian Pascu

**Affiliations:** 1 Laser Department, National Institute for Laser, Plasma and Radiation Physics, 409 Atomistilor, 077125 Magurele, Ilfov, Romania; mihai.boni@inflpr.ro (M.B.); angela.staicu@inflpr.ro (A.S.); mihai.pascu@inflpr.ro (M.L.P.); 2 Faculty of Physics, University of Bucharest, 405 Atomistilor, 077125 Magurele, Romania

**Keywords:** ciprofloxacin, laser degradation, fluorescence, HPTLC densitometry, FTIR, absorption spectroscopy

## Abstract

Ciprofloxacin is one of the most prescribed antibiotics in treating bacterial infections, becoming an important pollutant of the wastewaters. Moreover, ciprofloxacin is hard to be destroyed by conventional water treatment processes; therefore, efficient treatments to destroy it are needed in water decontamination. This study offers insights into the performance of 266 nm laser beams on the photodegradation of ciprofloxacin. An Nd:YAG laser was used that emitted 266 nm at an energy of 6.5 mJ (power of 65 mW) and ciprofloxacin water solutions were irradiated up to 240 min. The irradiated solutions were investigated by UV-Vis and FTIR absorption spectroscopy, pH assay, and laser-induced fluorescence. An HPTLC densitometer was used to characterize the laser-induced fluorescence and fluorescence lifetime of photodegradation products. The UV-Vis absorption, FTIR, and laser-induced fluorescence spectra showed the degradation of ciprofloxacin. Moreover, HPTLC densitometry offered the fluorescence and fluorescence lifetime of ciprofloxacin and its three photoproducts as well as their relative quantification. From the FTIR spectra, the molecular structure of two out of three photoproducts was proposed. In conclusion, the laser irradiation method provided the efficient photodegradation of ciprofloxacin, whereas the analytical techniques offered the proper means to monitor the process and detect the obtained photoproducts.

## 1. Introduction

Fluoroquinolone antibiotics, like ciprofloxacin (CIP), are overused in the veterinary, agricultural, and healthcare industries and are detectable in any aquatic environment [1]. Moreover, antibiotics that are released into the environment induce transcriptional changes in microbial communities, resulting in the development of antimicrobial resistance by microorganisms. CIP is a broad-spectrum antibiotic, intensively used primarily for treating Gram-negative bacterial-caused infections such as those for urinary tract infections, intra-abdominal infections, respiratory tract infections, bone and joint infections, or even skin infection [2]; thus, it is included in the World Health Organization’s List of Essential Medicines [3]. It kills bacteria by inhibiting bacterial DNA-gyrase and DNA topoisomerase, thus affecting DNA replication [4]. In 2018, CIP was prescribed more than six million times, making it the 109th most commonly prescribed drug in the United States [5].

Fluoroquinolone antibiotics are hard to be destroyed during conventional water treatment processes [6,7,8] and efficient wastewater treatment processes are needed to overcome the lack of methods in water decontamination. In this respect, CIP UV-Vis absorption spectrum shows an absorption maximum around 280 nm, making UV radiation the best choice in inducing CIP direct photolysis. As a consequence, it was observed that after being exposed to UV, the CIP solution loses its antibacterial activity [9].

Despite all conducted research, few methods are used to treat the water without reagents and out of these methods, the majority use lamps. The vacuum ultraviolet was used to photolyze water into hydroxyl radicals to induce the degradation of CIP, showing that in the early stage the hydroxylated intermediates were formed [10]. Moreover, in Reference [11], CIP was exposed to sunlight and UV radiation between 1 and 7 h emitted by UV lamps with powers of 15 W and 30 W. It resulted that, based on bacterial zone of inhibition assay, exposure to 30 W lamp for 6 h was optimal for the photodegradation of CIP. Ferguson et al. used three types of lamps (UVB: 290−350 nm/L, 5 J/cm^2^, UVA: 320−430 nm/L and 10 J/cm^2^, and Xenon arc solar simulator: 977 J/cm^2^) to degrade a sample of CIP in plasma and observed a decrease in antimicrobial activity of CIP when UVB radiation was used [9].

The study in this paper focused on the use of pulsed UV radiation (266 nm) emitted by a laser to speed up the photolysis process due to the optical characteristics of the laser radiation compared to that of a lamp.

Further, the identification and quantification of unknown compounds from a mixture are some of the fundamental problems in analytical chemistry. Analytical methods such as LC-MS/MS, GS-MS, and HPTLC have allowed one to investigate drugs at trace concentrations but present a series of disadvantages such as time-consuming experiments, high cost of analysis, or large quantities of samples. We propose a hyphenated technique, where high-performance thin-layer chromatography (HPTLC) is used to separate CIP and its photoproducts and spectroscopy methods provide analytical data for their characterization. The technique is an open-box concept offering the possibility to inspect the plate during scanning. Berman and Zare introduced laser-based detection methods for HPTLC in 1975, where subnanograms of aflatoxins were identified by laser-induced fluorescence (LIF) [12]. Since then, laser-based techniques have been developed to characterize the compounds separated via HPTLC, such as laser-based photoacoustic densitometer [13] or photothermal deflection densitometer [14].

This study aimed to induce the photolysis of CIP in water solution without adding reagents and to monitor its photolysis by UV-Vis and FTIR absorption spectroscopy, pH assay, LIF, and a laboratory-made HPTLC densitometer. The HPTLC densitometric system measured the LIF and fluorescence lifetime of the separated photoproducts. Further, it provided the chromatograms of the HPTLC plate.

## 2. Results

CIP solution at a concentration of 2 mg/mL in water was irradiated with a 266 nm laser beam having 6.5 mJ energy, up to 240 min and the degradation process was monitored at 1, 15, 30, 60, 120, 180, and 240 min by UV-Vis absorption spectroscopy, LIF spectroscopy, FTIR absorption spectroscopy, and HPTLC densitometry.

### 2.1. UV-Vis Absorption Spectroscopy

**Figure 1:**
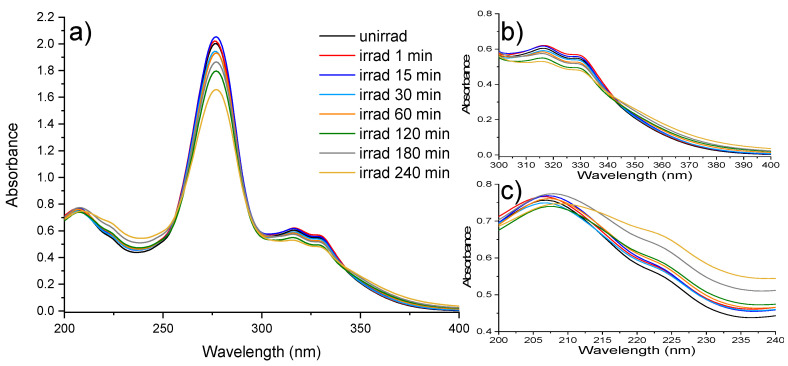
Absorption spectra of unirradiated and irradiated CIP for 1, 15, 30, 60, 120, 180, and 240 min, diluted to 0.2 mg/mL and recorded between (**a**) 200–400 nm, (**b**) 300–400 nm, (**c**) 200–240 nm.

Five absorption bands with peaks at 207 nm, 223 nm, 277 nm, 316 nm, and 331 nm characterized the absorption spectrum of CIP, which is depicted in Figure 1a. Due to CIP irradiation with 266 nm laser beam, the band with a peak at 277 nm suffered a hypochromic shift of 10.5% after 120 min of irradiation. The spectrum of 180 min irradiated sample showed a 3.7% increase in 277 nm peak absorbance compared with the sample irradiated at 120 min. Moreover, at the end of the 240 min irradiation, the absorption spectra showed a decrease in peak intensity of 11.1% compared with the 180 min irradiated CIP.

For the absorption bands with maxima at 316 nm and 331 nm in Figure 1b, an increase in intensity was observed in the first minute of irradiation, followed by a decrease until 120 min. Afterward, once again for 180 min irradiated CIP, a slight increase in intensity followed by a decrease for the 240 min irradiated CIP was observed. The decrease in absorbance peak is attributed to the photodegradation of CIP as observed in Reference [15].

As for the absorption band with a peak at 207 nm (Figure 1c), there was in the first 15 min of irradiation an increase in the intensity of 2.5%, followed by a decrease of 5.4% for CIP irradiated for 30 min compared with CIP irradiated for 15 min and of 2.9% compared with the unirradiated CIP. No bathochromic shifts of the absorption band were observed in the first 60 min of irradiation. Afterward, a bathochromic shift of 1 nm was observed for each remaining exposure time (120, 180, and 240 min), reaching a total shift of 3 nm at the end of the 240 min irradiated CIP.

**Figure 2:**
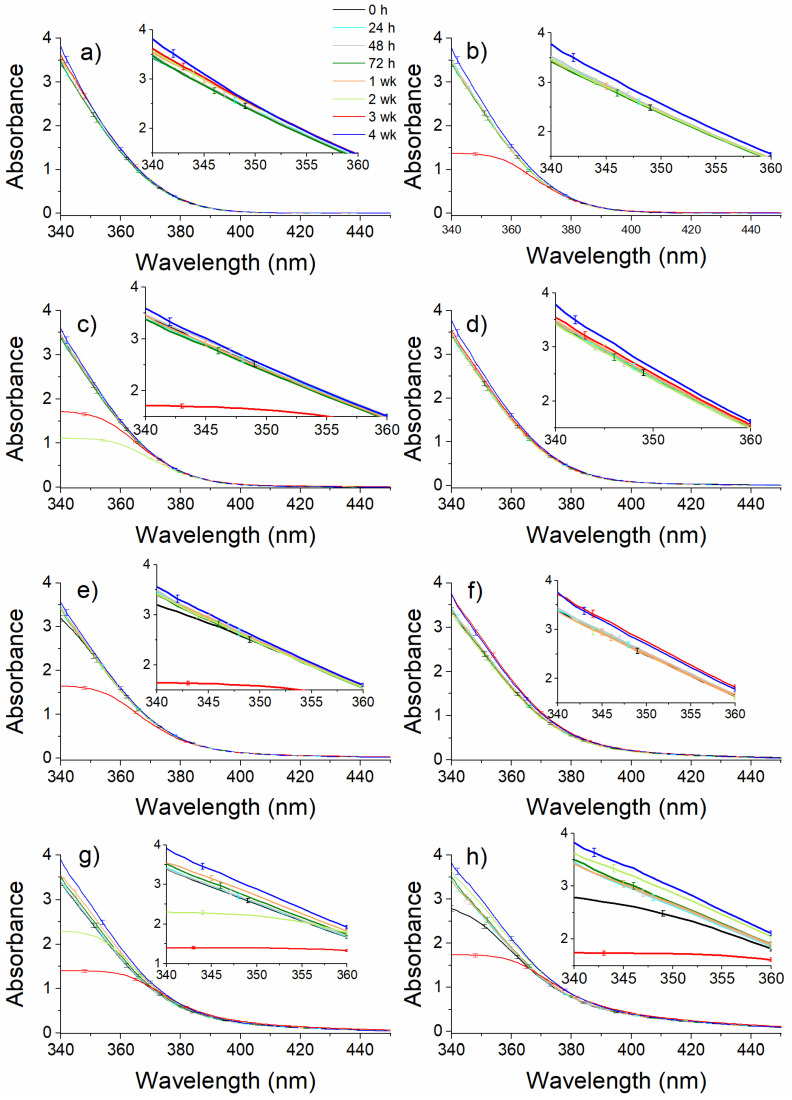
Stability studies over a 4-week time interval performed by recording UV-Vis absorption spectroscopy for CIP (**a**) unirradiated, (**b**) 1 min irradiated, (**c**) 15 min irradiated, (**d**) 30 min irradiated, (**e**) 60 min irradiated, (**f**) 120 min irradiated, (**g**) 180 min irradiated, (**h**) 240 min irradiated; the spectra were recorded at the initial concentration of 2 mg/mL.

Stability studies over a 4-week time interval were conducted by recording UV-Vis absorption spectra, immediately (0 h), at 24 h, 48 h, 1 week, 2 weeks, 3 weeks, and 4 weeks after the end of each irradiation process (Figure 2). CIP samples were kept in the dark at a temperature of 4 °C.

This study performed for the unirradiated CIP solution suggests that the sample was stable for 72 h. Irradiated CIP solutions for 1 min, 60 min, and 120 min remained stable for 2 weeks, and that exposed for 15 min was stable for 4 weeks. CIP irradiated for 30 min was stable for 3 weeks, and CIP solutions exposed to laser radiation for 180 min and 240 min were stable for 48 h and less than 24 h, respectively.

### 2.2. PH Assay

The assay was performed in triplicate and showed a decrease in pH during irradiation from 4.09 for unirradiated CIP to 2.99 for 240 min irradiated CIP. The pH values for each tested exposure interval are the following: unirradiated—4.09 ± 0.01, 1 min irradiated—3.88 ± 0.01, 15 min irradiated—3.8 ± 0.03, 30 min irradiated—3.75 ± 0.01, 60 min irradiated—3.47 ± 0.01, 120 min irradiated—3.35 ± 0.01, 180 min irradiated—3.1 ± 0.01, 240 min irradiated—2.99 ± 0.01.

### 2.3. Laser-Induced Fluorescence Assay

**Figure 3:**
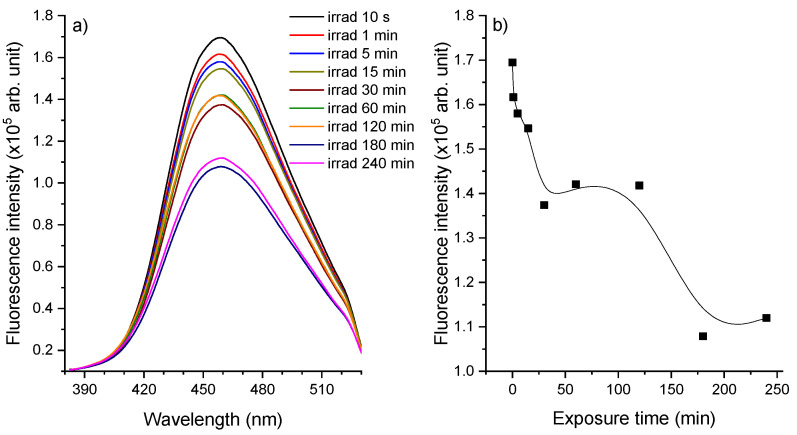
(**a**) LIF spectra of CIP, recorded in real-time during irradiation, in the 350–750 nm spectral range; (**b**) the fluorescence intensity of the 458 nm fluorescence band as a function of the laser irradiation time interval.

The LIF dispersed spectrum of 2 mg/mL CIP solution exposed for 240 min to a laser beam emitted at 266 nm was characterized by the presence of a single band with a peak at 458 nm (Figure 3a). During irradiation, the fluorescence peak suffered a hypochromic shift of 65% by the end of the 240 min. This decrease was not linear with irradiation time and changes are emphasized in Figure 3b.

Factors that can influence the LIF spectrum are pH and irradiation time. The decrease in pH during irradiation could affect the molecular reconfiguration that occurs following the protonation of the π electron system of the fluorophore [16].

As observed in Section 2.2, pH analysis showed a decrease during irradiation from 4.09 (unirradiated CIP) to 2.99 (240 min irradiated CIP), thus suggesting that pH could enhance the photodegradation process [17] and induce changes in fluorescence spectra. The behavior of fluorescence with irradiation time indicated the degradation of CIP into photoproducts and supported the results observed in the absorbance spectra.

### 2.4. FTIR Absorption Spectroscopy

**Figure 4:**
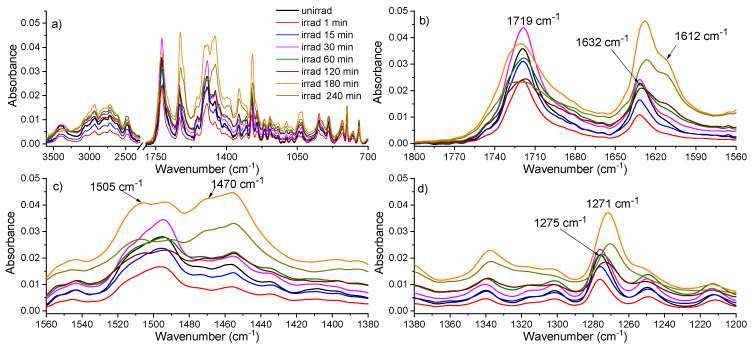
IR spectra of 2 mg/mL CIP unirradiated and irradiated between 1 min and 240 min in (**a**) 3600–700 cm^−1^, (**b**) 1800–1560 cm^−1^, (**c**)1560–1380 cm^−1^, (**d**) 1380–1200 cm^−1^ spectral range.

Unirradiated and irradiated CIP solutions were analyzed by FTIR spectroscopy and the vibrations corresponding to different bonds of the molecule were identified. The IR spectrum of unirradiated CIP was compared to those of irradiated solutions and the spectra are shown in Figure 4. Figure 4a presents the comparison between FTIR spectra of unirradiated and irradiated TZ solutions, whereas Figure 4b–d are closeups of regions of interest.

For unirradiated CIP, the characteristic vibrations are: 3390 cm^−1^—N–H stretching vibration (piperazinyl), 3057 cm^−1^—stretching C–H stretching vibration (aromatic ring), 2928, 2796, and 2721 cm^−1^—C–H stretching vibration (aliphatic), 2619, 2489, and 2469 cm^−1^—–NH_2_^+^ stretching vibration, 1719 cm^−1^—C=O stretching vibration (carboxyl), 1632 cm^−1^—C–O stretching vibration (quinoline), 1495 cm^−1^—C–C and C–N bending vibrations (quinoline), 1456 cm^−1^—C–N stretching vibration, 1301 cm^−1^—–CH_2_ bending vibration, 1275 and 1211 cm^−1^—C–N asymmetrical stretching vibration (piperazine), 1200–1140 cm^−1^—C–O and C–C stretching vibration, 1103–1020 cm^−1^—–CH_2_ bending vibration, 944–804 cm^−1^—–CH bending vibration (phenyl), and 779–745 cm^−1^—C–O–C bending vibration.

The disappearance of the 1275 cm^−1^ band from the IR spectra of the irradiated samples 120, 180, and 240 min suggests the cleavage of piperazine [18]. After 120 min irradiation, a new band appeared at 1271 cm^−1^ as observed in Figure 4d. In the case of irradiated CIP solutions between 15 min and 240 min, the band at 1612 cm^−1^ increases in intensity (Figure 4b), this being explained by the fact that the ratio between the maximum at 1632 cm^−1^ and the one at 1612 cm^−1^ decreased from 2.57 for unirradiated CIP to 1.26 for irradiated CIP at 240 min. This aspect indicated the stretching vibration C=O within a ketone which can be formed (Figure 4b).

For the irradiated samples at 120 min, 180 min, and 240 min, two bands appear at 1505 cm^−1^ and 1470 cm^−1^ assigned to the stretching vibration N–H (secondary amine), respectively the bending vibration (scissoring) –CH_2_ (Figure 4c). Moreover, the band with a peak at 1719 cm^−1^ presented an increased absorbance with irradiation time and an enlarged full width at half maximum (FWHM) after 120 min irradiation, as well. This is attributed to the stretching vibration of C=O, thus suggesting the formation of carboxyl.

### 2.5. HPTLC Densitometry

This method was developed in our laboratory [19] as an alternative to commercially available densitometers, and it is based on the detection of steady-state and time-resolved fluorescence of separated photoproducts when excited with a 375 nm picosecond diode laser. HPTLC densitometry was chosen because there was no solvent interference with the UV detection [20].

**Figure 5:**
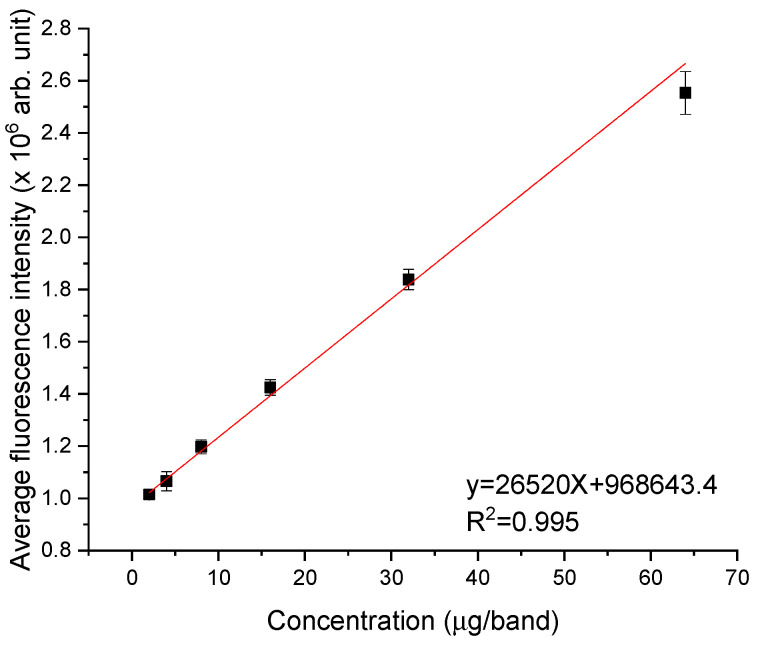
Calibration curve for CIP solutions at concentrations between 2 and 64 µg/band. The parameters of the fitting curve (red line) are indicated in the figure.

The linearity was investigated for three HPTLC plates, where on each plate were applied seven concentrations using the semi-automated equipment Linomat 5. The amount of CIP applied on each plate was 2, 4, 8, 16, 32, and 64 µg/band. The fluorescence spectra were recorded by point-by-point laser scan, following the left-to-right direction with an increment of 1 mm, obtaining in total, 100 fluorescence spectra. For each spectrum, corresponding to a given distance, the intensity of fluorescence peak was extracted. The next step was to plot the intensity of fluorescence peak as a function of distance, thus obtaining the horizontal chromatogram of CIP at various concentrations. Then, the fluorescence peak intensity of each concentration was plotted as a function of concentration (Figure 5) and the linear regression approach was used to determine the slope, the intercept, and the correlation coefficient for the calibration curve.

It was observed that in the concentration range 2–64 µg/band, the calibration curve is linear, with an average correlation coefficient of 0.993 ± 0.001. The relative standard deviation (%RSD) in this case was 0.22%. The %RSD is frequently used in method validation assays since it normalizes the standard deviation to the average [21] and the acceptance criteria is a %RSD smaller than 2%, as recommended by ICH guidelines [22].

One-way ANOVA statistical analysis was performed with a level of significance set at 0.05 and Fisher’s least-significant-difference (LSD) test was used to compare the differences between the mean values of fluorescence intensity peak extracted from the three chromatograms. The results of the semi-empirical assessment are shown in Table 1, where the sample means for the three chromatograms data sets were compared to each other.

The result of the analysis was F(2, 17) = 0.0947, F_critical_ = 3.68, and *p* = 0.91. The *p*-value suggested a high level of confidence and the null hypothesis was confirmed by the data. The F value of 0.0947 was lower than the F_critical_ value of 3.68, suggesting that the null hypothesis was not rejected. Thus, the F, F_critical_, and *p* values suggested that the means of data sets were not statistically different for a significance level of 0.05.

The compounds found in a developed HPTLC plate are dispersed along its band uniformly due to the heterogeneous distribution of silica gel [23]. Moreover, considering that the atmospheric conditions when developing a plate can vary (e.g., relative humidity), one can suggest that each plate should be regarded as a “new” chromatographic system [23]. Thus, the differences in average fluorescence could be due to sample distribution and sample application (volume error along with plate error, positioning error, and measurement error are less than 1.8%).

**Figure 6:**
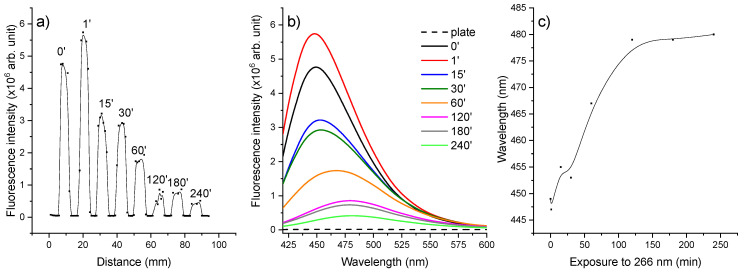
(**a**) Horizontal chromatogram resulting from point-by-point scanning of the plate before development in the mobile phase. (**b**) Fluorescence spectra of CIP and each irradiated CIP solution applied on the HPTLC plate. (**c**) Evolution of fluorescence peak wavelength with the irradiation time.

Furthermore, the unirradiated and irradiated CIP solutions were applied on an HPTLC plate that was developed, visualized at 254 nm using a TLC viewing cabinet, and photographed. Next, the steady-state and time-resolved fluorescence measurements were performed. The HPTLC plate was investigated before and after the development in the mobile phase. Figure 6 shows the LIF curves before the plate development: the bands (compounds) are found at the starting line and the photoproducts are not separated.

From the horizontal chromatogram, it was observed that the most intense fluorescence is attributed to a 1 min irradiated CIP solution (Figure 6a). Likewise, Figure 6b shows the different spectral characteristics of the irradiated solutions. Moreover, unirradiated and irradiated CIP solutions are differentiated both by the fluorescence intensity and by the wavelength of the fluorescence peak (Figure 6c). The unirradiated solution evidenced a fluorescence peak at 449 nm whereas the 240 min irradiated CIP had the peak at 480 nm.

**Figure 7:**
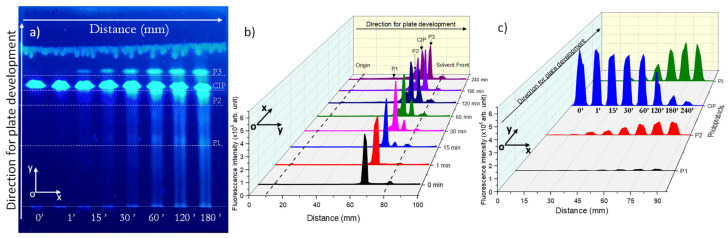
(**a**) Developed HPTLC plate, containing unirradiated CIP and the separated photoproducts of CIP, visualized at 254 nm and photographed. (**b**) Vertical (oy) chromatogram of the plate, (**c**) Horizontal (ox) chromatogram of the plate.

After developing, the HPTLC plate was visualized at 256 nm using a UV cabinet and then was photographed (Figure 7a). The separation of the photoproducts was observed. Three photoproducts were visualized and analyzed. The vertical chromatogram was obtained by selecting the center of each line and scanning the plate on y-component, thus resulting in the exact position of CIP and its photoproducts (Figure 7b). Next, each position of the compounds was selected and the x-component scanning was performed to determine the horizontal chromatogram (Figure 7c).

Figure 7b shows that the method can also detect the starting point (origin) and the solvent front of the developed HPTLC plate, thus helping in the determination of the retention factor (Rf) of each compound. The Rf is used to calculate the movement of the compounds along the plate and represent the distance covered by the compounds divided by the distance traveled by the mobile phase. The obtained Rf values were: P1—0.42, P2—71, CIP—0.78, and P3—0.85.

Better visualization of CIP degradation and the formation of its photoproducts is presented in Figure 7c, where it is observed that the fluorescence intensity is decreasing with prolonged exposure of CIP, which suggests its degradation into photoproducts. An increasing trend in fluorescence is observed for the photoproducts. P1 was observed after 30 min of irradiation, P2 was observed after 15 min of irradiation, and P3 after the first minute of irradiation.

**Figure 8:**
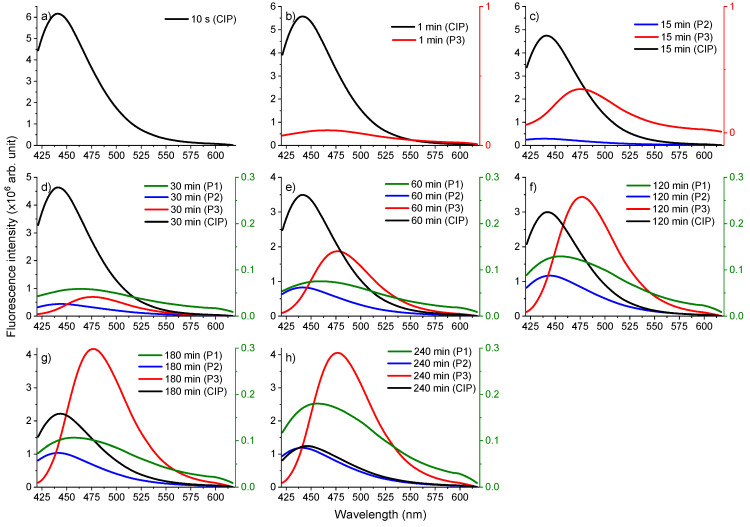
Time evolution of fluorescence spectra of CIP and its photoproducts for (**a**) 10 s, (**b**) 1 min, (**c**) 15 min, (**d**) 30 min, (**e**) 60 min, (**f**) 120 min, (**g**) 180 min, (**h**) 240 min irradiation. The right coloured oy axes correspond to the fluorescence curves of the compounds P3 (red), P1 (green).

Next, the spectral characteristics of the photoproducts were investigated and are shown in Figure 8. The wavelength of the fluorescence peak was: P1—477 nm, P2—486 nm, and P3—493 nm.

As observed in Figure 8, up to 60 min irradiation, the highest fluorescence belonged to CIP, but after 120 min irradiation, P3 presented the highest fluorescence intensity. The fluorescence intensity of P1 and P2 increased during irradiation, having a maximum after 240 min. In the case of P3, the fluorescence intensity increased until the sample was irradiated for 180 min, then it was followed by a decrease for the sample irradiated for 240 min. Therefore, the generation of photoproducts is not linear with the irradiation time and there is the possibility that not all the photoproducts resulted from CIP but from each other, as well.

**Figure 9:**
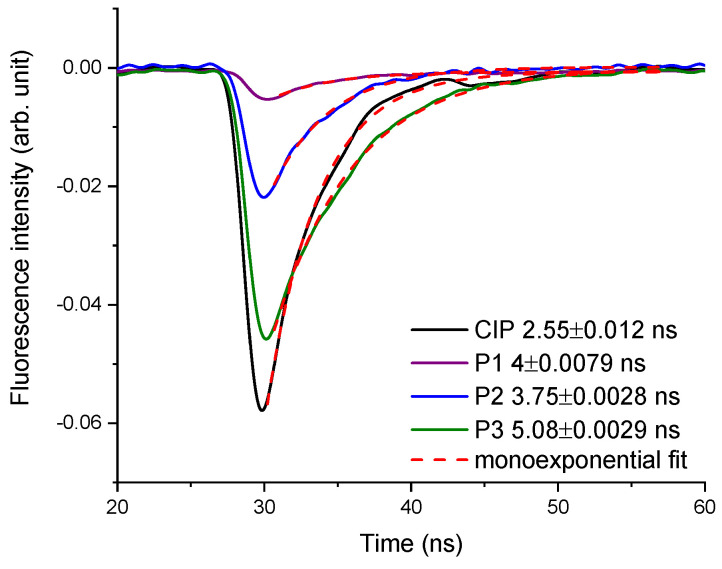
The time-resolved fluorescence signal of CIP, P1, P2, and P3 when excited at 375 nm.

As for the time-resolved fluorescence analysis, Figure 9 represents the time-resolved fluorescence signal for CIP and its photoproducts.

From the kinetics of the fluorescence signal, the values of the fluorescence lifetime were extracted using the mono-exponential fitting function. The lifetime value for CIP was 2.55 ns, whereas its photoproducts were: P1—4.08 ns, P2—3.75 ns, and P3—5.08 ns.

## 3. Discussion

The design of the assays reported in this paper was intended to evaluate the photodegradation of CIP exposed to UV-pulsed laser beams. The use of UV-Vis and FTIR absorption spectroscopy, LIF, and HPTLC densitometry proved to be useful techniques in optical characterization of the photoproducts formed during laser exposure.

As observed in the UV-Vis spectra, the presence of isosbestic points after 15 min irradiation suggested that new photoproducts were formed during laser exposure, with overlapping absorption bands, these photoproducts being in equilibrium [24]. Moreover, the modification of absorption intensity, absorption bands, and fluorescence intensity suggested the degradation of CIP.

The unirradiated CIP applied on the HPTLC plate presented a fluorescence peak at 449 nm (Figure 6), whereas the CIP water solution was characterized by a peak at 458 nm (Figure 3). Thus, a blue shift of 9 nm was observed between the CIP in water solution and CIP applied on the plate due to the interaction between each surrounding environment with the CIP molecule. The ultrapure water is a hydrogen-bonding solvent that affects the peak wavelength by altering the energy levels of non-bonding electrons and electrons in π* orbitals [25] and rigid polymer matrices such as HPTLC plate hinder the motion of the molecules [26]. It is probable that –O–Si–O– chains in the plate act as a barrier between CIP molecules to reduce their collisions and reduce the possibility of aggregation [27].

The disappearance of the band at 1275 cm^−1^ (assigned to C-N stretching vibration) and the increase in the intensity of the bands from 1612 cm^−1^ and 1719 cm^−1^(C=O stretching vibration) in the FTIR spectra suggest the formation of the CIP-2 photoproduct from Table 2 Moreover, the appearance of 1505 cm^−1^ and 1470 cm^−1^ bands characteristic of the stretching vibration N–H (secondary amine) and the bending vibration (scissoring) -CH_2_ (Figure 4c) indicates the CIP-3 photoproduct from Table 2. These molecular structures have to be confirmed in further studies by HPLC-MS. 

The piperazine moiety partial break that leads to the formation of CIP-2 and CIP-3 was also observed in other studies that involved the photodegradation of CIP under UV light [28,29,30].

**Figure 10:**
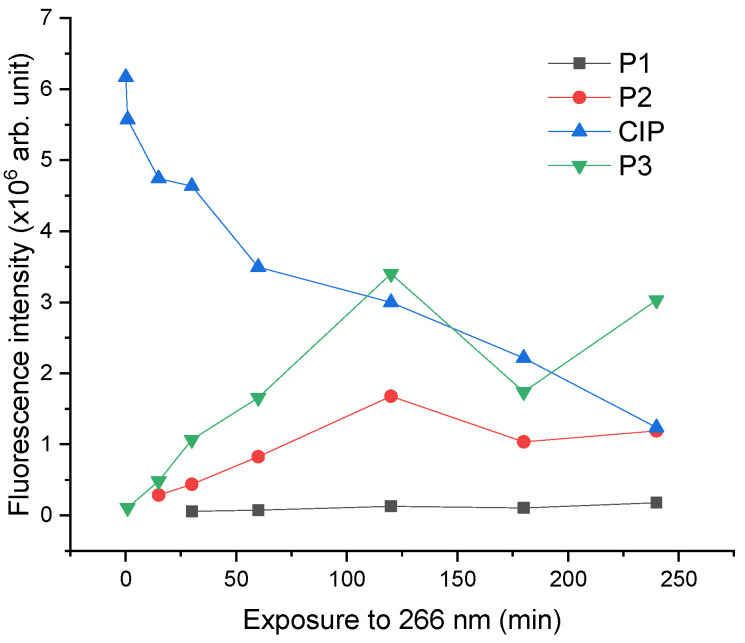
Fluorescence intensity peak evolution of CIP and its photoproducts during laser irradiation resulted from the HPTLC densitometry measurements.

Furthermore, the HPTLC densitometry assay was able to allow a useful comparison of the photoproducts and CIP side-by-side, where similarities and differences can be observed between LIF and fluorescence lifetime studies. Correspondingly, the relative quantification was obtained for CIP and each photoproduct (Figure 10).

Figure 10 shows the continuous photodegradation of CIP during the irradiation process, as its fluorescence intensity dropped by 80% at the end of 240 min of exposure. The initial concentration of CIP was 2 mg/mL and as shown in linearity assessment (Figure 5), this concentration was in the fluorescence linearity domain; thus, the quantitative determination was possible. It was calculated that the remaining quantity of CIP after the 240 min irradiation was 0.4 mg/mL. For the photoproducts, only the relative quantitative assessment can be made due to the lack of standards for which it is necessary to know the photoproducts and prepare stock solutions. P1 had an increase in fluorescence of 67.4% by the end of 240 min. Both P2 and P3 presented the highest fluorescence intensity for CIP irradiated for 120 min with an increase of 96.5% and 98.3%, respectively. Afterward, P2 fluorescence intensity decreased with 29.1% for CIP irradiated for 240 min compared to that of CIP irradiated for 120 min. As for P3, its fluorescence intensity decreased with 48.9% for CIP irradiated for 180 min compared with that obtained for CIP irradiated for 120 min, followed by an increase of 42.6% for CIP irradiated for 240 min compared with that of CIP irradiated for 180 min.

For the HPTLC densitometry, the pulsed laser diode was used due to its spectral irradiance compared with conventional UV lamps, and so, we were able to transfer more molecules in the excited state and produce a large number of photons over a short time (ps) allowing time-resolved studies. Fluorescence was chosen because it is useful for the quantitative determination of molecules at trace levels and it is one of the affordable techniques used to achieve a low limit of detection due to its high sensitivity and large linear dynamic range [31,32]. The advantages of the hyphenated technique are that there is no need to use two beams, one for reference and one for the sample, as in the commercially available densitometers, because we scan the entire lane having both the fluorescence of the reference (plate) and sample. The advantages of HPTLC densitometry over HPLC are simultaneous sample analysis, short system equilibrium time, lower costs, and the possibility of performing multiple scans of the same plate.

**Figure 11:**
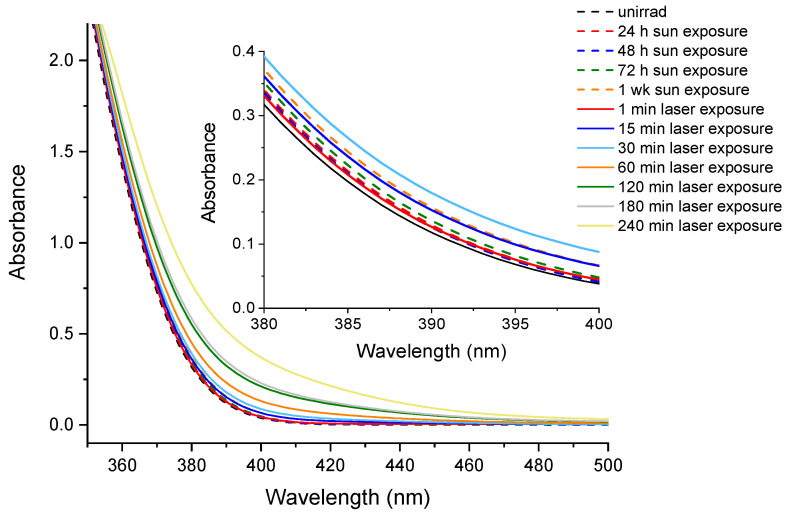
Absorption spectra for CIP exposed one week to sunlight and exposed 240 min to 266 nm laser radiation.

Moreover, to compare the effect of laser irradiation to that of sunlight, CIP was exposed in a quartz cuvette to sunlight for 1 week. The absorption spectra were collected at various time intervals as follows: 24 h, 48 h, 72 h, and 1 week. The comparison of absorption spectra between CIP exposed to laser radiation and sunlight is presented in Figure 11.

It was observed that only 1 min of laser exposure was necessary to obtain the same results as for 48 h sunlight exposure. Moreover, the modification in absorption spectra for the 15 min irradiation of CIP was more pronounced than that for one week CIP exposure to sunlight. These findings showed the importance of using a UV laser source to irradiate CIP to enhance its photodegradation.

## 4. Materials and Methods

### 4.1. Irradiation Protocol

CIP hydrochloride powders (WAK-Chemie Medical GmbH, 99.5% purity) were dissolved in ultrapure water at a concentration of 2 mg/mL. Volumes of 2 mL of sample were used in 1 cm optical path spectrophotometric cells and stirred continuously at 700 rpm to homogenize the sample. Irradiation of the compounds was performed for different exposure times, respectively 1, 15, 30, 60, 120, 180, and 240 min. The samples were irradiated with a laser beam emitted at 266 nm (6.5 mJ energy) by an Nd:YAG laser (Excel Technology, Surelite II model, FWHM 6 ns and 10 Hz pulse repetition rate). The cross-section area of the beam at the interface with the cuvette was 0.38 cm^2^, the fluence was 17.1 mJ/cm^2^ and the intensity was 171 mW/cm^2^.

### 4.2. Sample Characterization

#### 4.2.1. Laser-Induced Fluorescence

For laser-induced fluorescence (LIF) studies, the fluorescence was recorded in real-time and collected with an optical fiber (core diameter 1500 µm) positioned on the cuvette at 90° with respect to the incident beam. The spectra were recorded using a spectrograph (-SpectraPRO SP-2750, Acton Research, Trenton, NJ, USA) coupled to an ICCD camera (Princeton Instruments, model PIMAX 1024RB, Trenton, NJ, USA,).

#### 4.2.2. UV-Vis Absorption Spectroscopy

Absorption spectra were recorded between 200 and 450 nm using a Perkin Elmer spectrophotometer, Lambda 950 model, at a resolution of 1 nm. The used spectrophotometric cell had a 1 mm optical path. The spectra were also recorded for 0.2 mg/mL dilutions of the 2 mg/mL samples (initial concentration) to avoid obtaining a saturated signal in the spectral range of 200–360 nm.

#### 4.2.3. PH Assay

The samples’ pH was measured with an accuracy of ±0.01 using Schott Instruments Lab 860 pH-meter (BlueLine 16pH electrode).

#### 4.2.4. FTIR Absorption Spectroscopy

The IR spectrum was recorded using a Nicolet T iS ™ 50 FTIR spectrometer, in the spectral range 4000–750 cm^−1^, at a resolution of 4 cm^−1^, and an average of 32 spectra. The CIP samples were dried on KRS-5 support, applying 20 µL of the liquid sample, and the KRS-5 spectrum was subtracted from the final spectrum.

#### 4.2.5. HPTLC Densitometry

The high-performance thin-layer chromatography (HPTLC) plate used in the experiment was an Alugram Nano-Sil G (Roth) aluminum plate precoated with 0.2 mm silica gel, size 10 × 10 cm. The plates were prewashed in methanol and activated at 100 °C for 30 min. Irradiated CIP samples were applied to the HPTLC plate using the Linomat 5 semi-automatic system (CAMAG). A volume of 4 µL of the solution was applied to the plate as a band (5 mm) at a dosing rate of 20 nl/s. The mobile phase consisted of a mixture of dichloromethane: methanol: 25% ammonia (4/2/0.85, *V/V/V*). After the plate was developed, it was allowed to dry and then it was visualized and photographed in UV light at 256 nm using the Chromo-Vue^®^ Cabinet C-65 (UVP) chamber.

The HPTLC densitometer consisted of a laser diode (Alphals, PicoPower LD-37550, 375 nm, 30 MHz, pulse duration of 87.7 ps, measured power of 490 µW), automated XY stage (8MTF-102LS05, Standa), optical fiber (core diameter 1500 µm, 300–1200 nm), spectrograph (Acton Research model, SpectraPRo SP-2750, Trenton, NJ, USA), UV-Vis-NIR photomultiplier (Hamamatsu H-6780-02, 300–850 nm, Hamamatsu City, Japan), and oscilloscope (Tektronix DPO 7254, Beaverton, USA). A dielectric mirror (BB1-E01, ThorLabs) was used to direct the laser beam on the plate. The signal recorded by the spectrograph or the oscilloscope was triggered by a TTL signal provided from the laser source. The fiber was placed at 45° to the incident beam.

To measure the LIF signal, the output of the fiber was coupled to the spectrograph and LIF spectra were collected with an increment of 1 mm in both OX and OY directions. The fluorescence peak intensity of each spectrum was extracted and plotted as function of the distance traveled by the laser beam on the HPTLC plate, thus resulting in the chromatograms. For fluorescence lifetime studies, the output of the fiber was coupled to the photomultiplier whose output signal was coupled to the digital oscilloscope. Because the fluorescence lifetime is independent of CIP concentration, the signals were collected when the laser beam was situated in the center of the compound band from the HPTLC plate. The spectra were fitted with a monoexponential decay function and resulted in the fluorescence lifetimes.

## 5. Conclusions

The study reported in this paper constitutes a potentially successful method for CIP removal from water. Laser-induced fluorescence could assist to monitor in real-time the degradation of CIP and offline methods such as UV-Vis absorption spectroscopy, FTIR spectroscopy, and HPTLC densitometry could confirm the removal of CIP. The experimental setup can be adjusted in terms of laser beam area or fluence, to offer the best removal conditions and thus larger volumes of water could be treated. Further studies must be conducted where the selectivity of the method should be tested for a mixture of antibiotics, for instance.

## 6. Patents

A patent application no. A/00120 from 18.03.2021 was submitted to the State Office for Inventions and Trademarks, Romania.

## Figures and Tables

**Table 1:** Variance (ANOVA) statistical analysis followed by Fisher’s LSD test of the fluorescence intensity peaks extracted from the chromatograms of the three HPTLC plates.

Comparison between Plates	Mean Diff.	SEM	Statistical Significance	LCL	UCL
Plate 2 ↔ Plate 1	25534.7	342196.8	No	−703840.5	754909.9
Plate 3 ↔ Plate 1	−114304.5	342196.8	No	−843679.8	615070.7
Plate 3 ↔ Plate 2	−139839.2	342196.8	No	−869214.5	589535.9

Mean Diff.: difference between the means of two compared data sets; SEM: standard error of the mean; LCL: lower confidence limits; UCL: upper confidence limits.

**Table 2:** Proposed molecular structure of photodegradation products resulting from laser irradiation of CIP.

CIP	CIP-2	CIP-3
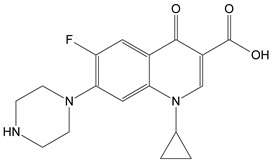	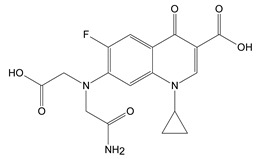	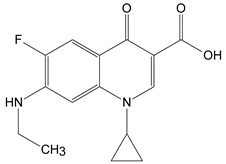

## Data Availability

Data available on request.

## Sample Availability

Samples of the compounds TZ and irradiated TZ ... are available from the authors.
